# Supplementary material for: All-optical nonequilibrium pathway to stabilising magnetic Weyl semimetals in pyrochlore iridates
Source: Nat Commun. 2018 Oct 26;9:4452. doi: 10.1038/s41467-018-06991-8 (PMC6203748; doi:10.1038/s41467-018-06991-8)
Supplement: Supplementary file 1 — Supplementary Information [file 41467_2018_6991_MOESM1_ESM.pdf]

Supplementary Information for Topp et al., All-optical nonequilibrium pathway to stabilizing magnetic Weyl semimetals in pyrochlore iridates

Gabriel E. Topp,[1] Nicolas Tancogne-Dejean,[1] Alexander F. Kemper,[2] Angel Rubio,[1,3] and Michael A. Sentef*[1]

[1]*Max Planck Institute for the Structure and Dynamics of Matter, Center for Free Electron Laser Science, 22761 Hamburg, Germany*

[2]*Department of Physics, North Carolina State University, Raleigh, NC, USA*

[3]*Center for Computational Quantum Physics (CCQ), Flatiron Institute, 162 Fifth Avenue, New York NY 10010*

(Dated: October 2, 2018)

*Correspondence and requests for materials should be addressed to M.A.S. by email: michael.sentef@mpsd.mpg.de

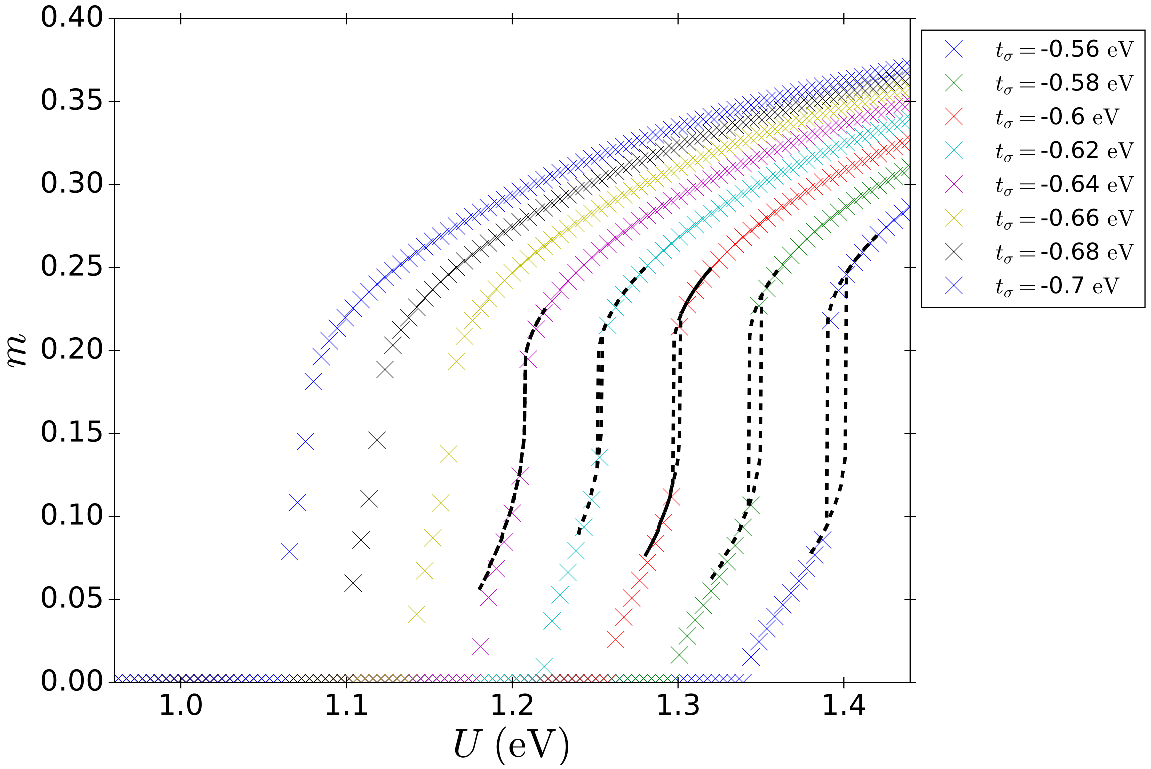

Supplementary Figure 1. **Computed ground state magnetization.** **a**, Self-consistently calculated magnetic order parameter as a function of Hubbard U for different hopping values t_σ, varied in steps of 0.02 eV from -0.56 eV to -0.70 eV at a finite temperature, $T = 0.016$ eV. Starting from $t_\sigma = -0.62$ eV, with increasing hopping an increasing region of hysteresis is found, indicating a first-order phase transition.

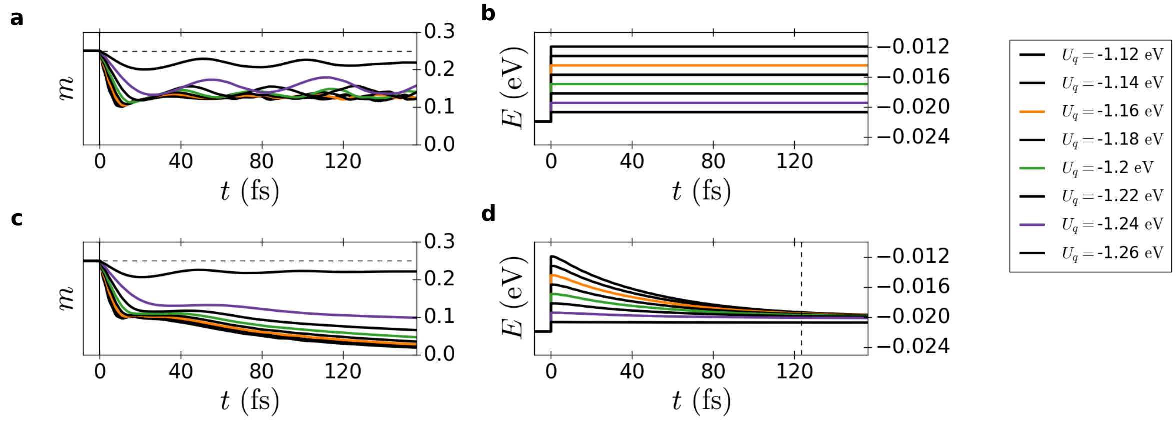

Supplementary Figure 2. **Nonequilibrium magnetization and energy.** **a**, Closed system ($\Gamma_0 = 0$) time evolution of the magnetic order parameter for different quench values U_q, varied in steps of 0.02 eV within the interval $[1.12, 1.26]$ eV. **b**, Nonequilibrium energy per particle per unit cell for the corresponding interaction interval. The system energy only changes at $t = 0$, at which the (closed) system is quenched. The amount of energy pumped into the system scales linearly with ΔU. It remains constant before and after the quench. The final energy values are used to calculate the effective nonequilibrium temperatures T_eff, displayed in Figure 2(c) in the main text. **c**, Magnetization dynamics of the open system ($\Gamma_0 = 0.008$ eV) for same parameter set. **d**, Time-dependent total energy of the open system. After the quench at $t = 0$, the total energy relaxes towards its thermal steady-state value on a time scale $\Gamma_0^{-1} \approx 80$ fs. The dashed vertical line denotes the probe time, $t_\mathrm{p} = 123.4$ fs, used for Figure 3(b-d) of the main text.

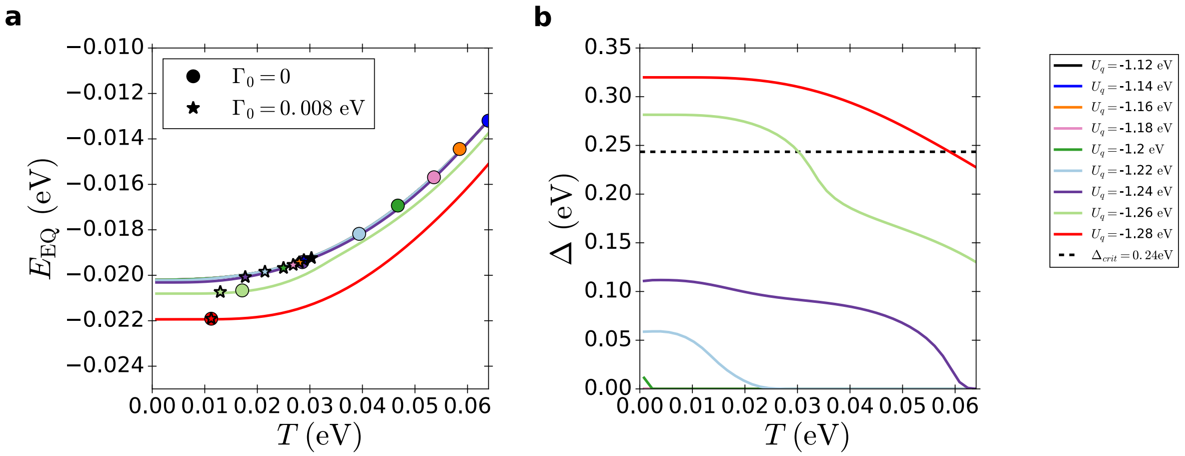

Supplementary Figure 3. **Temperature dependence of total energy and magnetization.** **a**, Temperature-dependent equilibrium mean energy per particle per unit cell for different values U, varied in steps of 0.02 eV within the interval $[1.12, 1.28]$ eV. From these curves, the effective temperatures T_{eff}, assigned to the nonequilibrium states in Figure 2(b, d), can be read off by attributing each energy value at the probe time, $t_{\text{p}} = 123.4$ fs, to its corresponding temperature. **b**, $\Delta \equiv U \cdot m$ in dependence of the equilibrium temperature. The WSM-PMM phase boundary can be read off at that point where $\Delta(T)$ reaches zero. $\Delta_{\text{crit}} \approx 0.24$ eV denotes the critical value at which the AFI-WSM transition takes place. The dip appearing for intermediate interactions is associated with the semi-metallic band structure and change in density of states in the WSM phase as opposed to the insulating AFI phase.

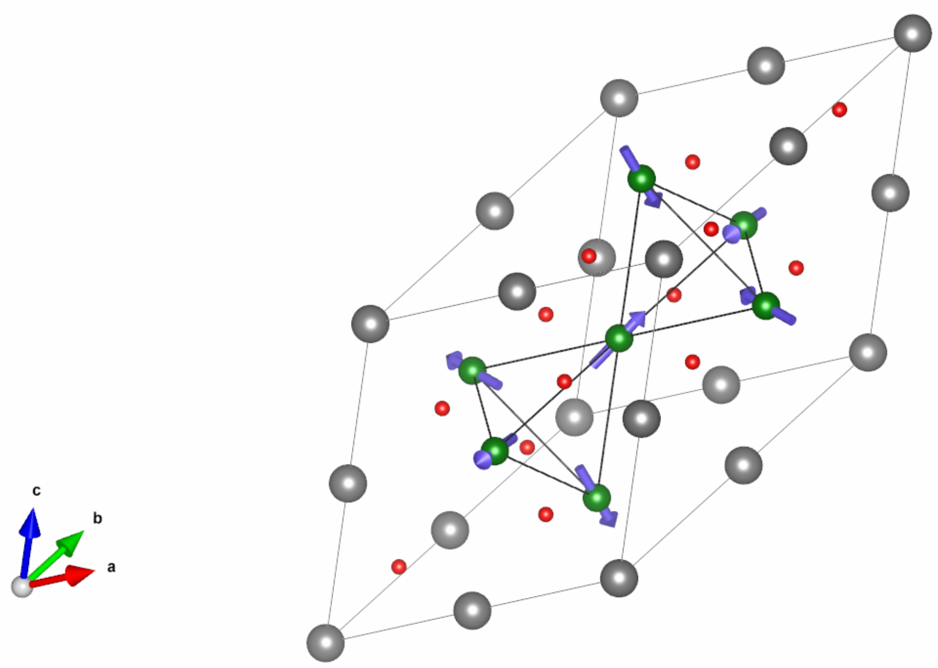

Supplementary Figure 4. ***Ab initio* magnetization configuration.** All-in all-out magnetic configuration (blue arrows) obtained for $Y_2Ir_2O_7$, computed from the density matrix of the localized $5d$ orbitals of iridium atoms (green spheres).
