# Supplementary material for: All-optical nonequilibrium pathway to stabilising magnetic Weyl semimetals in pyrochlore iridates
Source: Nat Commun. 2018 Oct 26;9:4452. doi: 10.1038/s41467-018-06991-8 (PMC6203748; doi:10.1038/s41467-018-06991-8)
Supplement: Supplementary file 3 — Description of Additional Supplementary files [file 41467_2018_6991_MOESM3_ESM.pdf]

Video - Movie of nonthermal Weyl semimetal
